# Supplementary material for: Evaluation of Foot and Claw Lesions and Claw Horn Growth in Piglets from Birth to End of Nursery
Source: Animals (Basel). 2023 Nov 10;13(22):3477. doi: 10.3390/ani13223477 (PMC10668767; doi:10.3390/ani13223477)
Supplement: Supplementary file 1 [file animals-13-03477-s001.zip › animals-2644340-supplementary.pdf]

animals

MDPI

Article

Evaluation of Foot and Claw Lesions and Claw Horn Growth in Piglets from Birth to End of Nursery

Maren Bernau [1,2,*], Hannah Meckel [1,3], Theresa Dölle [1] and Armin Manfred Scholz [1]

Table S1: Bruising score results, differentiated for the different genetic lines, days of life and claw locations.

				score bruising																		Fisher's exact test *
				GL						GL-LW						Pi-PiDu						
d (n)	claw location			0	1	2	3	4	5	0	1	2	3	4	5	0	1	2	3	4	5	
0	front	*dorsal*	inner	79.92	15.38	7.69				93.33	3.33	3.33				91.67	4.17	4.17				0.0136
(67)			outer	92.31	7.69					86.67	10.00	3.33				95.83	4.17					0.0571
		palmar	inner	61.54	30.77	7.69				100						58.33	8.33	33.33				<0.0001
			outer	53.85	30.77	15.38				96.67	3.33					54.17	25.00	20.83				<0.0001
	rear	*dorsal*	inner	100						100						95.03	4.17					0.3582
			outer	92.31	7.69					100						100						0.1940
		palmar	inner	69.23	30.77					96.67	3.33					75.00	8.33	16.67				<0.0001
			outer	92.31	7.69					96.67	3.33					75.00	12.50	12.50				0.0029
3	front	*dorsal*	inner	78.95	15.79	5.26				100						100						0.0048
(67)			outer	84.21	10.53	5.26				92.31	7.69					100						0.0181
		palmar	inner	31.58	10.53	26.32	26.32	5.26		26.92	42.31	19.23	11.54			17.39	34.78	39.13	8.70			<0.0001
			outer	15.79	31.58	36.84	15.79			30.77	30.77	34.62	3.85			8.70	30.43	47.83	13.04			<0.0001
	rear	*dorsal*	inner	73.68	26.32					80.77	19.23					91.30	8.70					0.0266
			outer	78.95	15.79	5.26				88.46	11.54					91.30	8.70					0.0230
		palmar	inner	15.79	47.37	26.32	10.53			26.92	38.46	11.54	23.08			4.35	65.22	21.74	8.70			<0.0001
			outer	21.05	47.37	15.79	15.79			42.31	23.08	26.92	7.09			30.43	52.17	17.39				<0.0001
8	front	*dorsal*	inner	68.42	26.32	5.26				91.67	8.33					95.83	4.17					0.0028
(67)			outer	47.37	42.11	10.53				87.50	12.50					91.67	8.33					<0.0001
		palmar	inner	5.26	10.53	26.32	36.84	15.79	5.26	20.83	12.50	33.33	25.00	8.33		8.33	4.17	29.17	37.50	20.85		<0.0001
			outer	5.26	0	31.58	47.37	10.53	5.26	12.50	12.50	25.00	50.00			8.33	4.17	33.33	25.00	29.17		<0.0001
	rear	*dorsal*	inner	63.16	31.58	5.26				75.00	20.83	4.17				100						<0.0001
			outer	57.89	36.84	5.26				79.17	16.67	4.17				100						<0.0001
		palmar	inner	5.26	15.79	21.05	36.84	15.79	5.26	20.83	20.83	16.67	25.00	12.50	4.17	4.17	25.00	29.17	33.33	8.33		<0.0001
			outer	21.05	15.79	21.05	42.11			12.50	25.00	20.83	25.00	16.67		12.50	29.17	33.33	20.83	4.17		<0.0001
28	front	*dorsal*	inner	89.47	10.53					65.22	26.09	8.70				77.27	13.64	4.55	4.55			0.0014
(64)			outer	89.47	10.53					69.57	17.39	13.04				81.82	18.18					0.0029
		palmar	inner	26.32	31.58	5.26	36.84			39.13	17.39	26.09	13.04	4.35		22.73	59.09	18.18				<0.0001
			outer	31.58	26.32	21.05	21.05			34.78	43.48	17.39	4.35			31.82	45.45	18.18	4.55			<0.0001
	rear	*dorsal*	inner	73.68	15.79	5.26	5.26			69.57	21.74	8.70				72.73	27.27					0.0020
			outer	89.47	5.26	5.26				39.13	39.13	13.04	8.70			77.27	13.64	9.09				<0.0001
		palmar	inner	36.84	15.79	31.58	10.53	5.26		52.17	30.43	13.04	4.35			18.18	68.18	13.64				<0.0001
			outer	68.42	21.05	5.26	5.26			65.22	17.39	12.04	4.35			31.82	40.01	22.73	4.55			<0.0001
71	front	*dorsal*	inner	78.95	15.79	5.26				55.56	22.22	22.22				90.48	4.76	4.76				<0.0006
(58)			outer	73.68	26.32					83.33	11.11	5.56				85.71	14.29					0.0151
		palmar	inner	63.16	26.32	10.53				44.44	27.78	27.78				66.67	28.57	4.76				0.0010
			outer	52.63	21.05	21.05	5.26			50.00	16.67	27.78	5.56			71.43	14.29	9.52	0	4.76		<0.0002
	rear	*dorsal*	inner	68.42	26.32	5.26				66.67	22.22	11.11				85.71	14.29					0.0045
			outer	52.63	36.84	10.53				38.89	33.33	22.22	5.56			85.71	4.76	9.52				<0.0001
		palmar	inner	47.37	36.84	15.79				50.00	16.67	22.22	11.11			19.05	66.67	14.29				<0.0001
			outer	31.58	26.32	26.32	10.53	5.26		16.67	16.67	61.11	5.56			52.38	33.32	9.52	4.76			<0.0001

GL = German Landrace; GL-LW = crossbred of German Landrace boar and Large White sow; Pi-PiDu = crossbred of Piétrain boar and Pí étrain-Durocsow. **Results of Fisher´s exact text using Monte Carlo estimation.*

Table S2: Claw lesion score results, differentiated for the different genetic lines, days of life and claw locations.

			GL					GL-LW					Pi-PiDu					Fisher's exact
d (n)	location		0	1	2	3	4	0	1	2	3	4	0	1	2	3	4	test *
0	front	inner	100					73.33	26.67				4.17	58.33	37.50			<0.0001
(67)		outer	69.23	30.77				43.33	43.33	13.33			0	41.47	58.33			<0.0001
	rear	inner	92.31	7.69				60.00	26.67	13.33			4.17	45.83	50.00			<0.0001
		outer	76.92	23.08				43.33	36.67	20.00			0	45.83	54.17			<0.0001
3	front	inner	57.89	31.58	10.53			34.62	46.15	15.38	3.85		100					<0.0001
(67)		outer	36.84	52.63	10.53			7.69	50.00	38.46	3.85		100					<0.0001
	rear	inner	57.89	21.05	21.05			23.08	42.31	34.62			100					<0.0001
		outer	42.11	42.11	15.79			3.85	50.00	46.15			100					<0.0001
8	front	inner	41.11	57.89				12.50	37.50	45.83	4.17		8.35	87.50	4.17			<0.0001
(67)		outer	21.05	78.95				0	54.17	45.83			0	83.33	16.67			<0.0001
	rear	inner	57.89	42.11				20.83	45.83	33.33			12.50	66.67	20.83			<0.0001
		outer	47.37	47.37	5.26			8.33	54.17	37.50			12.50	66.67	20.83			<0.0001
28	front	inner	52.63	42.11	5.26			78.26	17.39	4.35			31.82	40.91	13.64	9.09	4.55	<0.0001
(64)		outer	42.11	52.63	5.26			65.22	30.43	4.35			31.82	50.00	4.55	13.64		<0.0001
	rear	inner	47.37	47.37	5.26			60.87	34.78	4.35			27.27	36.36	18.18	18.18		<0.0001
		outer	31.58	68.42				65.22	30.43	4.35			27.27	40.91	18.18	13.64		<0.0001
71	front	inner	89.47	10.53				100					33.33	66.67				<0.0001
(58)		outer	84.21	15.79				100					47.62	52.38				<0.0001
	rear	inner	89.47	10.53				94.44	5.56				47.62	47.62	4.76			<0.0001
		outer	94.74	0	5.26			94.44	5.56				57.14	38.10	4.76			<0.0001

GL = German Landrace; GL-LW = crossbred of German Landrace boar and Large White sow; Pi-PiDu = crossbred of Piétrain boar and Piétrain-Duroc sow. *Results of Fisher´s exact text using Monte Carlo estimation.
